# Linear Immunoglobulin A (IgA) Bullous Dermatosis Mimicking Stevens-Johnson Syndrome

**DOI:** 10.7759/cureus.30309

**Published:** 2022-10-14

**Authors:** Jason S Park, Caitlin D Hamilton, Shaan Patel, Jason B Lee, Sylvia Hsu

**Affiliations:** 1 Dermatology, Lewis Katz School of Medicine, Temple University, Philadelphia, USA; 2 Dermatology, Geisinger Commonwealth School of Medicine, Scranton, USA; 3 Dermatology, Thomas Jefferson University, Philadelphia, USA

**Keywords:** direct immunofluorescence, chronic bullous disease of childhood, autoimmune bullous disease, vancomycin, drug-induced, stevens-johnson syndrome (sjs), toxic epidermal necrolysis (ten), linear iga bullous dermatosis

## Abstract

Linear IgA bullous dermatosis (LABD) is a rare autoimmune blistering disease characterized by linear IgA deposition along the dermo-epidermal junction on direct immunofluorescence (DIF). LABD appears clinically as erythematous polycyclic lesions in younger patients but can show considerable phenotypic heterogeneity in older patients, often leading to misdiagnoses such as bullous pemphigoid, pemphigus vulgaris, Stevens-Johnson syndrome/toxic epidermal necrolysis (SJS/TEN), or other bullous conditions. Cases of LABD mimicking SJS/TEN require prompt skin biopsies for histopathology and DIF for disease differentiation and medical decision-making. In cases of suspected drug-induced LABD or SJS/TEN, identification and removal of the offending agent are paramount. The preferred treatment for LABD is oral dapsone, while SJS/TEN may respond better to cyclosporine or a combination of intravenous immunoglobulin and systemic corticosteroids. This case highlights the rare occurrence of LABD mimicking SJS/TEN and emphasizes the details that clinicians must know to guide patient management.

## Introduction

Linear IgA bullous dermatosis (LABD) is a rare autoimmune mucocutaneous disease affecting young children and adults and characterized by subepidermal blisters with linear IgA deposition along the dermo-epidermal junction (DEJ) on direct immunofluorescence (DIF) [1]. In children, LABD is also known as the chronic bullous disease of childhood and typically manifests on the lower abdomen, genitals, extremities, or around the mouth and eyes as polycyclic erythematous plaques with blistering along the borders ("string of pearls" sign) [2]. Conversely, adult-onset LABD displays considerable phenotypic heterogeneity and usually involves the trunk, head, and extremities [1].

Stevens-Johnson syndrome (SJS) manifests as rapidly coalescing erythematous macules which invariably progress to blistering and sheet-like desquamation of the skin and mucous membranes if left untreated. SJS and toxic epidermal necrolysis (TEN) lie on a clinical spectrum defined by body surface area involvement (<10% BSA: SJS, 10-30% SJS-TEN overlap, >30%: TEN) and represent true dermatologic emergencies [3, 4]. Histologically, SJS/TEN may show features (e.g., epidermal necrosis, orthokeratosis, sparse dermal inflammation) like those found in erythema multiforme or fixed drug eruption, making clinical correlation vital in these cases [4].

Cutaneous manifestations are often preceded by flu-like symptoms in SJS/TEN, while patients with LABD may complain only of itching or be completely asymptomatic [5, 6]. Involvement of the genital, respiratory, and gastrointestinal mucosa is more common in SJS/TEN than in LABD, but the cornea can be affected in both diseases [5]. Drug-induced LABD (DILABD) usually presents with more severe, atypical blistering that can mimic SJS/TEN and is more likely to show a positive Nikolsky sign when compared to idiopathic forms [6]. Herein we present a case of DILABD, which was initially misdiagnosed as SJS/TEN, and highlight key information that dermatologists should know about this relatively rare entity.

## Case presentation

**Figure 1:**
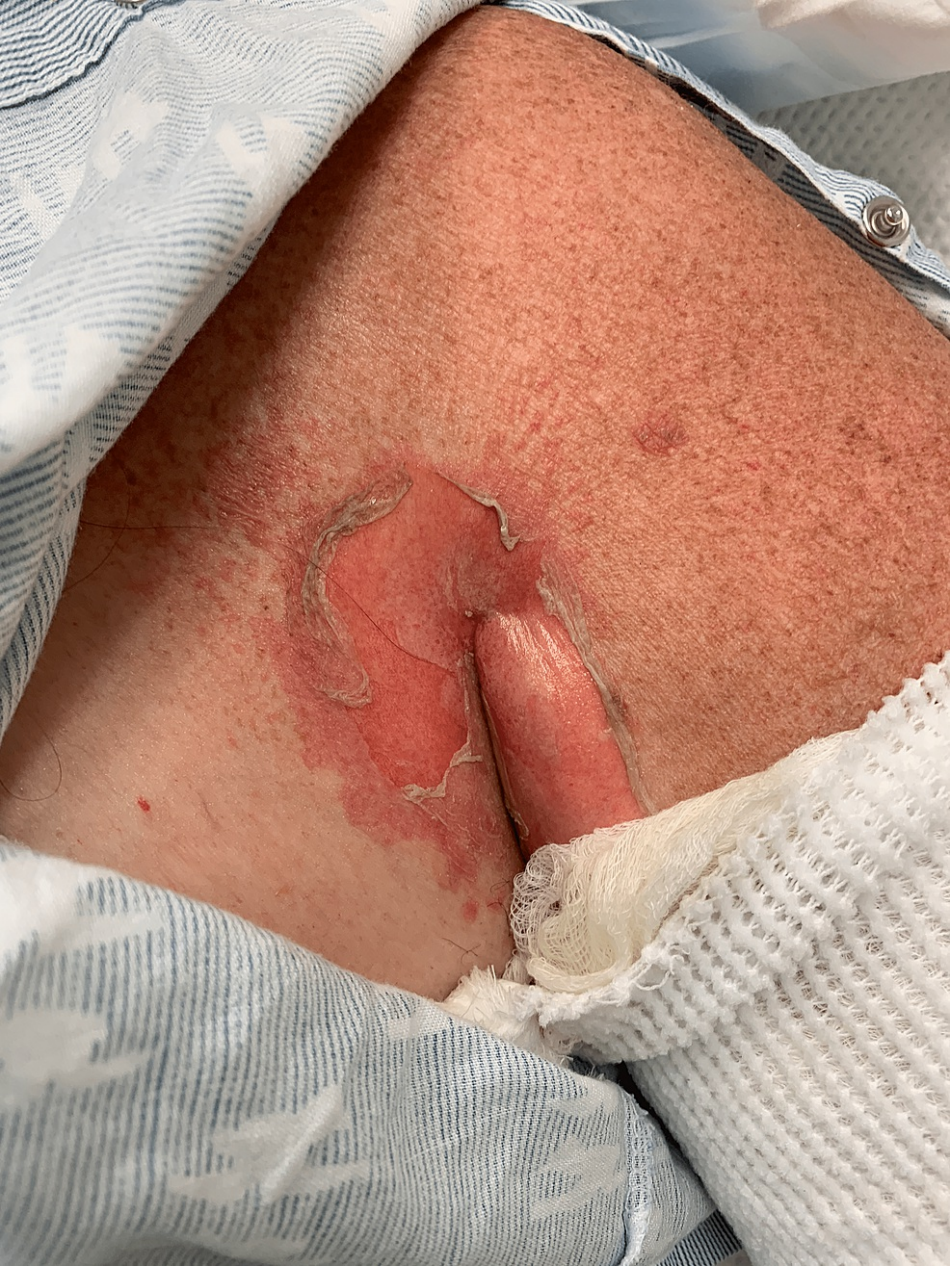
Sheet-like desquamation in the axilla

**Figure 2:**
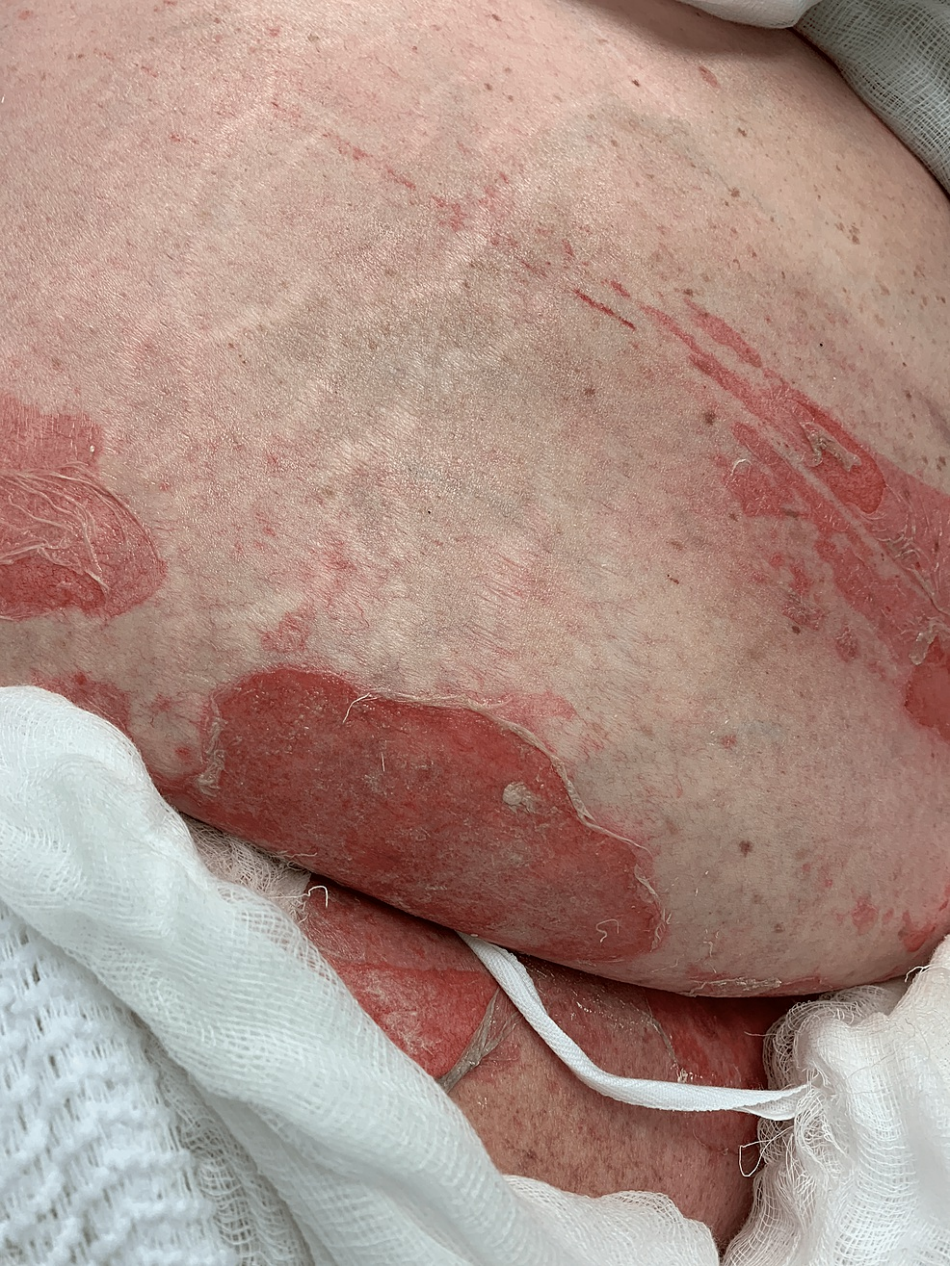
Sheet-like desquamation on the abdomen

A 66-year-old woman with a history of cardiorenal syndrome, chronic obstructive pulmonary disease, and hypothyroidism presented to the emergency department one day after developing erosions on her skin and in her mouth. Due to concern for SJS or TEN, methylprednisolone 40 mg was administered intravenously every eight hours and supportive measures were initiated. On day four, several areas of denuded skin were observed on the axillae (Figure 1), abdomen (Figure 2), inframammary, and inguinal folds, and within the oral cavity. Additionally, a single tense bulla on an erythematous base was found on the palm. Two punch biopsies were taken from the left axilla for hematoxylin and eosin (H&E) and DIF.

**Figure 3:**
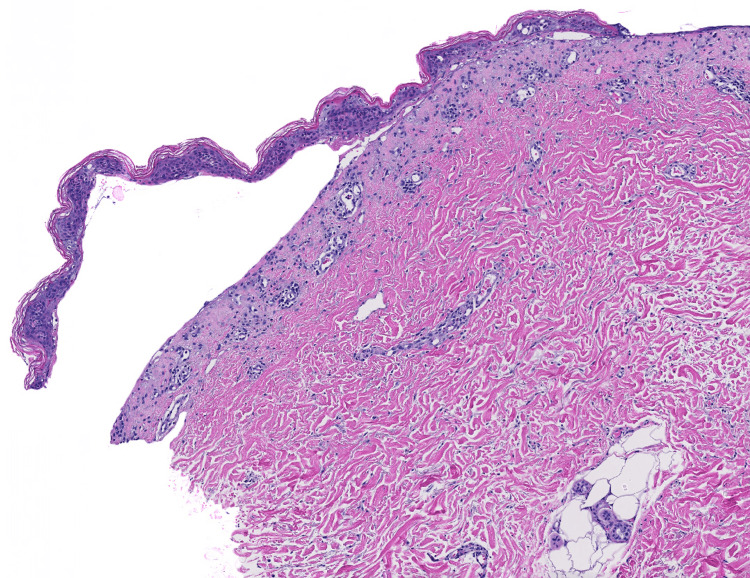
Pauci-inflammatory subepidermal blister with few lymphocytes, eosinophils, and neutrophils (H&E 40X).

H&E revealed a pauci-inflammatory subepidermal blister with few lymphocytes, eosinophils, and neutrophils (Figure 3). The DIF showed prominent linear IgA deposition at the DEJ. The second set of skin biopsies examined by an outside laboratory confirmed similar results. Indirect immunofluorescence (IIF) showed linear IgA antibody deposition on the epidermal side of salt-split skin. These findings were most consistent with a diagnosis of LABD.

Oral dapsone 100 mg/day was initiated, and the lesions improved gradually over the next two weeks. No new blisters or erosions were observed. The patient’s clinical course was complicated by pneumonia and acute hypoxemic respiratory failure, which ultimately led to her death seven weeks after the initial presentation. Medical records revealed that the patient had received one dose of intravenous vancomycin 10 days prior to the appearance of skin lesions.

## Discussion

LABD is often confused with other clinically similar diseases. In a retrospective study of 23 cases of LABD from Denmark, 16 cases of adult-onset LABD were initially misdiagnosed as bullous pemphigoid, dermatitis herpetiformis, SJS/TEN, eczema, bullous dermatitis not specified, erythema multiforme, bullous impetigo, acquired epidermolysis bullosa, or dermatophytosis [2]. Documented cases of idiopathic LABD or DILABD specifically mimicking SJS or TEN are sparse in the literature. There have been only a few reports in the past decade. Most recently, a TEN-like presentation of LABD was reported in a patient following a third dose of the Moderna COVID-19 vaccine [7].

Sporadic case reports and series of DILABD have also been described, with more than 40 different drugs linked to the disease [1, 2, 6]. However, vancomycin-induced LABD represents more than half of all reported cases [6] Furthermore, in a retrospective series of 69 cases of DILABD, vancomycin was the suspect drug in 57% of cases [1]. Cutaneous lesions typically manifest 2 to 21 days after vancomycin exposure and may take several weeks to resolve after drug cessation [6]. Phenytoin and trimethoprim/sulfamethoxazole are the next most common drugs implicated in DILABD after vancomycin [6]. Both of these drugs, along with several antibiotic classes (e.g. cephalosporins, sulfonamides, aminopenicillins) and non-steroidal anti-inflammatory drugs (NSAIDs), are associated with SJS/TEN as well, which can further delay the correct diagnosis. Interestingly, other anti-epileptic agents, including lamotrigine, carbamazepine, valproic acid, and phenobarbital, are commonly implicated in SJS/TEN but not in DILABD. [4, 6].

In idiopathic LABD, several dermal antigenic targets of IgA autoantibodies have been identified, including the 97-kDa (LABD97) and 120-kDa (LAD-1) fragments of bullous pemphigoid (BP) antigen 180, BP230, LAD285, laminin 332, and type VII collagen (COL7) [6, 8, 9]. In contrast, the pathomechanisms underlying DILABD are still being investigated [10]. Yamagami et al. recently demonstrated that IgA reactivity to the basement membrane zone was enhanced by the addition of vancomycin to patient serum in a dose-dependent manner on IIF and identified COL7 as a target antigen of IgA autoantibodies [8] Similarly, no unifying pathomechanism exists for SJS/TEN. However, some reports suggest T-cell mediated, type IV hypersensitivity or hapten/pro-hapten responses to drugs and infectious triggers [4].

To make the diagnosis of LABD, dermatologists should obtain skin biopsies for histopathology and DIF and interpret the microscopic findings within the clinical context. When SJS or TEN are part of the differential diagnosis, particularly in ill patients who initially present with prodromal flu-like symptoms, frozen sections may be preferred to expedite medical decision-making [4]. Management of DILABD requires prompt identification and withdrawal of the offending agent, which may be challenging in cases of polypharmacy. Oral dapsone with or without topical corticosteroids is considered first-line therapy based on case series and expert opinion [6]. In some reports, prednisone, sulfapyridine, colchicine, intravenous immunoglobulin (IVIG), and other immunomodulating drugs have been used with variable efficacy [1]. Conversely, cyclosporine is the preferred treatment for SJS while IVIG and systemic corticosteroids are most efficacious for treating SJS-TEN overlap and TEN along with supportive measures in an intensive care setting [4].

## Conclusions

Although idiopathic and DILABD are relatively rare, clinicians must consider this entity in cases of possible SJS/TEN and perform both H&E and DIF staining to make an accurate, timely diagnosis. Vancomycin is the most commonly implicated drug in DILABD. However, other antibiotics, anti-epileptic agents, and NSAIDs are frequently associated with DILABD and/or SJS/TEN as well. A detailed exposure history must be analyzed in conjunction with the clinical course and biopsy results to optimize patient management.
